# Aortic Stiffness Measured by Carotid Femoral-Pulse Wave Velocity at Different Stages of Normal Glucose, Prediabetes, and Diabetes Mellitus: A Systematic Review and Meta-Analysis

**DOI:** 10.31083/j.rcm2509339

**Published:** 2024-09-23

**Authors:** Xiao Liang, Dongdong Li, Zhen Wang, Yuxin Cheng, Ke Mou, Chenyu Ye, Yunyou Duan, Yong Yang

**Affiliations:** ^1^Department of Ultrasonic Diagnosis, Tangdu Hospital of Air Force Medical University, 710038 Xi’an, Shaanxi, China; ^2^Department of Cardiology, Tangdu Hospital of Air Force Medical University, 710038 Xi’an, Shaanxi, China

**Keywords:** aortic stiffness, carotid-femoral pulse wave velocity, diabetes mellitus, prediabetes, systematic review

## Abstract

**Background:**

To explore aortic stiffness measured by carotid femoral-pulse wave velocity (cf-PWV) at different stages of normal glucose, prediabetes, and diabetes mellitus (DM).

**Methods:**

The literature comparing aortic stiffness (AS) with cf-PWV between DM and non-DM samples was systematically retrieved from Pubmed, Ovid Medline, Web of Science, Embase, Scopus, CNKI, and Wanfang databases. The Newcastle–Ottawa Scale was used to assess the quality of the literature. The primary endpoint was the mean difference (MD) of cf-PWV between the normal glucose and DM samples and normal glucose and prediabetes samples. The secondary endpoints were the MD of carotid intima-media thickness (cIMT) and carotid-radial pulse wave velocity (cr-PWV). Aggregated MD and 95% confidence intervals were calculated. When the I^2^ value was >50% or *p* < 0.01, the heterogeneity was considered large, and the random-effect model was used; otherwise, the fixed-effect model was used. A sensitivity analysis was conducted to identify the source of heterogeneity, and a funnel plot and the regression Egger test was utilized to assess the publication bias.

**Results:**

A total of 37 studies were finally enrolled. Samples with DM had a higher cf-PWV value and cIMT value than those without DM, and the differences were statistically significant. The cr-PWV measurements tended to be higher in the DM group than in the non-DM group, but the difference was not significant. Samples with prediabetes also had a significantly higher cf-PWV value than samples with normal glucose.

**Conclusions:**

Samples with DM and prediabetes were associated with a higher cf-PWV value, indicating that DM patients had a higher central AS. Central AS progresses at the prediabetes stage. These data provide insight into understanding the mechanism of adverse effects of DM and prediabetes on artery stiffness.

## 1. Introduction

Diabetes mellitus (DM) is one of the most challenging public health problems worldwide, with an increasing prevalence and a wide range of complications affecting individuals, health systems, and national economies [1]. DM is also considered one of the independent risk factors of cardiovascular events and is closely related to aortic stiffness (AS), which is accelerated by DM [2, 3]. Prediabetes has also attracted much attention in recent years since hyperglycemia most likely occurs before the diagnosis of DM. A large number of studies have attempted to demonstrate the relationship between DM and AS, some of which also involve prediabetes. These studies have used different indicators, including carotid intima-media thickness (cIMT), augmentation index (AIx), and local or regional pulse wave velocity (PWV). Of these, regional PWV is currently assumed to be the most reliable method [4].

Regional PWV can be divided into carotid–femoral PWV (cf-PWV), carotid–radial PWV (cr-PWV), and brachial–ankle PWV (ba-PWV). cf-PWV reflects the stiffness of the central artery, cr-PWV reflects the peripheral artery, and ba-PWV reflects both central and peripheral arteries. Among them, cf-PWV has been regarded as the gold standard method for assessing AS and acts as a predictor of future cardiovascular disease and all-cause mortality [4, 5].

There are many potential physiological and pathological mechanisms in which hyperglycemia affects arterial stiffness. For example, the accumulation of advanced glycation end products in the DM arterial wall leads to cross-linking of collagen molecules and reduced collagen elasticity, resulting in increased arterial stiffness [6]. The state of insulin resistance before DM leads to excessive insulin, increased collagen, proliferation of smooth muscle cells, and eventually increased arterial stiffness [7]. In addition, impairment of endothelial function and a mild inflammatory response may partly explain the increase in arterial stiffness associated with hyperglycemia [8].

Information regarding the association between cf-PWV and DM has sometimes been contradictory: Most studies have shown that cf-PWV is increased in patients with DM compared with non-DM controls [2, 3, 9, 10, 11, 12, 13, 14, 15, 16, 17, 18, 19, 20, 21, 22, 23, 24, 25, 26, 27, 28, 29, 30, 31, 32, 33, 34, 35, 36, 37, 38, 39, 40, 41], although some other studies have shown no significant difference in cf-PWV [42, 43, 44]. The relationship between prediabetes and cf-PWV is also unclear. However, a meta-analysis has yet to be conducted to review the studies in this field. Therefore, this study sought to explore and clarify the correlation between DM or prediabetes and AS measured by cf-PWV to provide further insights for clinical practice. This study was registered on https://inplasy.com/(No. INPLASY2023110073).

## 2. Methods

### 2.1 Literature Searching Strategy

A systematic literature retrieval was conducted using the combined search terms “arterial stiffness”, “pulse wave velocity”, and “diabetes” in two Chinese databases—“CNKI” and “Wanfang” and five English databases—“Pubmed”, “Ovid MEDLINE”, “Embase”, “Web of Science”, and “Scopus”, from the inception of each database up to September 2023. The references of the obtained articles were also reviewed. Additional relevant articles were included if they met the inclusion criteria.

### 2.2 Literature Inclusion and Exclusion Criteria

Studies were enrolled if they met the following criteria: (1) studies comparing cf-PWV between DM and non-DM samples; (2) the study’s language was limited to either Chinese or English; (3) observational studies.

Studies were excluded if they met the following criteria: (1) studies with incomplete baseline information; (2) studies that were assessed as low quality or high-risk bias by two independent reviewers; (3) repetitive studies: studies with the same subjects published in different journals or different periods; (4) studies whose data could not be retrieved.

### 2.3 Endpoints and Definitions

Primary endpoints: cf-PWV, the propagation velocity of the pulse wave between the common carotid artery and the ipsilateral common femoral artery, measured by the relevant distance divided by the transmission time, reflects the aortic AS.

Secondary endpoints: cr-PWV and cIMT. cr-PWV, the propagation velocity of the pulse wave between the common carotid artery and the ipsilateral radial artery, measured by the relevant distance divided by the transmission time, reflects the peripheral artery AS. cIMT reflects the change in common carotid artery structure and was measured by ultrasound.

### 2.4 Data Extraction and Quality Assessment

XL and DL independently and rigorously collected the data characteristics from the enrolled literature. Conflicts were settled by discussion with ZW. The collected data included basic information on the studies and subjects and the results of cf-PWV, cr-PWV, and cIMT. XL and DL evaluated the quality of the enrolled studies using the Newcastle–Ottawa Quality Assessment Scale [45]. The full score was 9 points: 7 to 9 points were deemed high-quality studies, 4 to 6 were medium-quality studies, and less than 4 were low-quality studies.

### 2.5 Statistical Analysis

Stata/MP 17.0 (Lakeway Drive, College Station, TX, USA) was used to calculate the aggregated mean differences (MD) with 95% confidence intervals (CIs). The I^2^ test was performed to assess the heterogeneity among the studies. A random-effect model was used if *p* < 0.01 or I^2^ > 50%; otherwise, the fixed-effect model was used. The origin of heterogeneity was identified by conducting a sensitivity analysis. Publication bias was evaluated by funnel plots and an Egger test based on regression. A *p*-value less than 0.05 was deemed statistically significant.

## 3. Results

### 3.1 Searching Results and Baseline Information

**Fig. 1.**
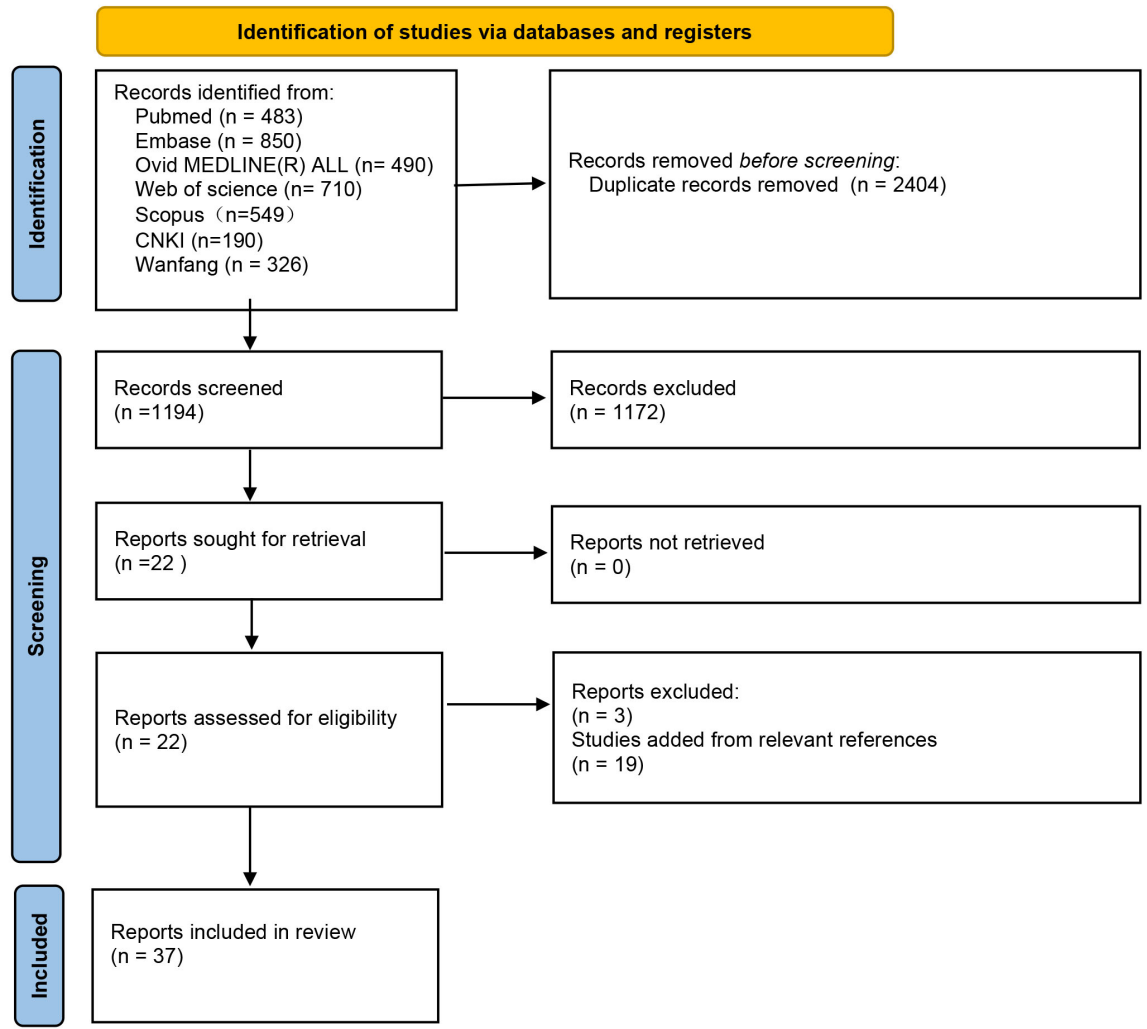
**Flowchart of the literature searching process**.

Pubmed, Ovid MEDLINE, Web of Science, Embase, Scopus, CNKI, and Wanfang databases were searched from inception to Sep. 2023. The search items and strategy are shown in Table 1. From the 3598 identified studies, 2404 were excluded for being duplicates. Of the remaining 1194 articles, 22 articles met the inclusion criteria (Full text all available), and 4 articles met the exclusion criteria (1 study with repetitive subjects and 3 studies with incomplete data), 19 articles were added from reviewing the references, and finally, 37 articles were included [2, 3, 9, 10, 11, 12, 13, 14, 15, 16, 17, 18, 19, 20, 21, 22, 23, 24, 25, 26, 27, 28, 29, 30, 31, 32, 33, 34, 35, 36, 37, 38, 39, 40, 41, 43, 44]. These included 21 English and 16 Chinese articles, 7 involving prediabetes, 4 involving type 1 diabetes (T1DM), 24 involving type 2 diabetes (T2DM), and 9 involving undefined diabetes classification. The flowchart for identifying these studies is shown in Fig. 1. The baseline information of the enrolled studies and participants is shown in Table 2 (Ref. [2, 3, 9, 10, 11, 12, 13, 14, 15, 16, 17, 18, 19, 20, 21, 22, 23, 24, 25, 26, 27, 28, 29, 30, 31, 32, 33, 34, 35, 36, 37, 38, 39, 40, 41, 43, 44]).

**Table 1.** **The searching strategies and searching results**.

Database	Searching strategy	
CNKI	(title: arterial stiffness(precise)) OR (title: pulse wave velocity (precise)) AND (title: diabetes(precise))	190
Wanfang	title: (“arterial stiffness”) or title: (“pulse wave velocity”) and title: (“diabetes”)	326
Embase	(‘arterial stiffness’:ti OR ‘pulse wave velocity’:ti) AND diabetes:ti	850
Pubmed	(“arterial stiffness”[Title] OR “pulse wave velocity”[Title]) AND “diabetes”[Title]	483
Ovid MEDLINE	(“pulse wave velocity”[Title] OR “arterial stiffness”[Title]) AND “diabetes”[Title]	490
Web of Science	(((TI=(arterial stiffness)) OR TI=(pulse wave velocity)) AND TI=(diabetes))	710
Scopus	( TITLE ( arterial AND stiffness ) OR TITLE ( pulse AND wave AND velocity ) AND TITLE ( diabetes ) )	549

**Table 2.** **The baseline information of the enrolled studies and participants**.

Study	Sample size	Age, years	Male, %	BMI, kg/m^2^	Smoker, %	Systolic pressure mmHg	Diastolic pressure, mmHg	Hypertension, %	Total cholesterol, mmol/l	HDL-cholestero, l mmol/L	LDL-cholesterol, mmol/L	Triacylglycerols, mmol/L	Dyslipidaemia, %	Heart rate, bpm	Fasting blood glucose, mmol/L	HbA1c, %	Creatinine, µmol/L	Country
	Non-DM/DM	Non-DM/DM	Non-DM/DM	Non-DM/DM	Non-DM/DM	Non-DM/DM	Non-DM/DM	Non-DM/DM	Non-DM/DM	Non-DM/DM	Non-DM/DM	Non-DM/DM	Non-DM/DM	Non-DM/DM	Non-DM/DM	Non-DM/DM	Non-DM/DM	
Bruno RM, *et al*. 2012 [2]	175/84	54.4/56.7	60.0/58.0	26.9/32.8	18.0/12.0	-	-	100.0/100.0	5.70/4.90	1.40/1.30	3.50/2.90	1.30/1.60	-	66.8/75.0	5.20/8.90	5.60/8.00	74.0/73.0	Italy
Zhang M, *et al*. 2011 [3]	79/79	60.1/60.2	49.0/49.0	24.4/24.7	43.6/36.2	119.3/123.1	72.2/72.9	-	4.65/5.05	1.49/1.32	2.60/3.05	1.09/1.51	-	73.4/77.6	4.88/6.97	-	69.1/61.4	China
Podgórski M, *et al*. 2019 [9]	50/50	13.1/13.4	50.0/52.0	22.6/23.5	-	119.0/113.0	73.0/67.0	-	-	-	-	-	-	-	-	-	-	Poland
Coutinho MN, *et al*. 2018 [10]	37/37	65.9/64.1	56.8/59.5	27.7/31.4	-	130.0/140.0	80.0/77.0	97.2/94.4	4.29/4.73	1.34/1.19	2.34/2.68	1.45/1.99	-	-	4.89/7.89	5.60/8.40	206.0/196.3	Brazil
Cameron JD, *et al*. 2003 [11]	112/57	71.2/61.5	54.5/68.4	-	-	137.4/144.6	79.0/84.0	-	-	-	-	-	-	-	-	-	-	England
Luneva EB, *et al*. 2022 [12]	27/37	54.0/57.5	44.0/46.0	35.6/32.9	41.0/32.0	130.0/131.0	81.0/77.0	70.0/57.0	5.40/4.84	1.15/1/11	3.49/2.67	1.88/2.58	-	-	-	5.74/8.90	-	Russia
Toyoshima MTK, *et al*. 2023 [13]	30/30	34.0/34.0	50.0/50.0	24.4/22.9	-	-	-	0.0/30.0	4.81/4.22	1.46/1.48	2.51/2.24	0.97/0.78	-	-		5.40/8.30	-	Brazil
Loehr LR, *et al*. 2016 [14]	849/1090	75.2/75.0	31.0/47.0	26.0/29.6	6.0/5.0	-	-	61.0/84.0	-	-	-	-	-	63.3/66.5	5.15/7.75	5.30/6.70	-	American
Dybjer E, *et al*. 2018 [15]	1539/553	71.8/72.9	35.7/47.9	-	8.6/9.2	141.4/147.4	-	-	5.49/4.64	-	-	-	-	66.5/69.2	5.46/7.77	-	-	Sweden
Gordin D, *et al*. 2016 [16]	25/46	58.8/62.5	-	27.3/33.0	4.0/9.0	-	-	-	5.30/4.20	1.30/1.40	3.60/2.40	1.30/1.60	-	-	-	5.40/7.60	-	Finland
Sciacqua A, *et al*. 2020 [17]	271/45	49.5/49.5	39.5/73.3	28.4/30.5	20.7/20.0	144.3/145.7	90.2/91.2	-	-	1.40/1.21	3.27/3.40	1.37/1.62	-	69.2/71.1	5.06/6.60	-	-	Italy
Tuttolomondo A, *et al*. 2019 a [18]	40/40	61.4/61.8	32.5/25.0	26.6/30.2	-	130.1/133.3	72.5/77.3	25.0/95.0	-	-	-	-	10.0/70.0	-	-	-/8.10	-	Italy
Tuttolomondo A, *et al*. 2019 b [18]	40/40	61.0/61.4	40.0/42.5	27.7/29.4	-	124.0/123.5	71.5/70.2	15.0/72.5	-	-	-	-	5.0/50.0	-	-	-/7.20	-	Italy
Wiromrat P, *et al*. 2019 [43]	61/169	15.3/15.4	43.0/41.0	-	-	107.0/113.0	64.0/69.0	-	3.76/4.17	1.24/1.35	2.07/2.38	0.85/0.87	-	-	-	5.30/9.00	61.9/56.6	American
Metsämarttila E, *et al*. 2018 [19]	244/119	68.5/68.5	36.0/52.0	25.9/29.7	-	141.0/144.0	77.0/78.0	-	5.50/5.00	1.80/1.50	3.50/3.20	-	-	-	5.40/6.20	5.69/6.26	-	Finland
Vallée A, *et al*. 2018 a [20]	78/44	53.7/57.8	59.0/74.5	25.5/27.0	51.3/47.7	124.0/127.0	75.0/79.0	0.0/0.0	-	-	-	-	10.2/36.4	68.0/72.0	-	5.30/7.70	86.7/86.1	Frence
Vallée A, *et al*. 2018 b [20]	232/160	61.3/63.4	59.5/61.9	27.4/29.7	38.3/41.3	137.0/139.0	77.0/78.0	100.0/100.0	-	-	-	-	26.7/63.8	67.0/73.0	-	5.60/7.40	77.5/75.8	Frence
van Mil SR, *et al*. 2018 [21]	157/43	39.8/47.4	21.7/41.9	42.7/42.7	24.8/27.9	138.0/143.0	81.0/82.0	49.0/72.1	-	1.20/1.20	3.30/2.50	1.70/1.96	79.0/79.1	-	-	5.63/7.73	-	Netherlands
Funck KL, *et al*. 2021 [22]	59/45	57.9/59.3	47.0/49.0	25.9/29.3	49.0/53.0	131.0/126.0	83.0/79.0	-	5.70/4.40	-	3.40/2.20	-	-	-	-	5.60/6.50	-	Denmark
Muhammad IF, *et al*. 2017 [23]	2382/532	71.9/72.7	-	-	9.8/8.5	-	-	-	-	1.47/1.28	3.43/2.84	0.90/1.10	-	62.6/64.3	5.70/7.80	-	-	Sweden
Martagón AJ, *et al*. 2022 [24]	161/420	44.0/53.5	31.7/40.0	27.8/28.9	-	110.0/114.0	71.0/74.0	-	4.82/5.05	1.19/1.09	2.95/3.04	1.31/2.15	-	-	5.11/7.06	5.40/7.50	61.9/61.9	Mexico
Zhou J, *et al*. 2018 a [44]	357/142	63.9/64.8	42.9/43.7	-	-	132.2/132.2	79.9/77.1	-	-	-	-	-	-	70.3/72.3	-	-	-	China
Zhou J, *et al*. 2018 b [44]	108/65	63.1/65.6	44.4/44.6	-	-	135.0/132.8	81.0/77.7	-	-	-	-	-	-	71.1/75.8	-	-	-	China
Tian CW, *et al*. 2015 [25]	60/60	64.6/66.9	50.0/50.0	26.3/24.3	-	134.4/133.5	81.9/78.6	-	5.30/5.53	1.30/1.27	2.86/2.95	1.53/1.97	-	-	5.34/7.40	-	-	China
Liu LJ, *et al*. 2021 [26]	72/107	56.3/58.5	38.9/59.8	-	-	-	-	-	-	-	-	-	-	-	-	-	-	China
Zhang JX, *et al*. 2011 [27]	30/40	47.5/46.7	56.7/55.0	25.1/25.3	-	112.5/139.7	80.3/80.8	-	4.99/5.16	1.56/1.43	2.15/2.68	1.24/2.25	-	-	4.98/9.27	5.15/8.13	-	China
Li CS, *et al*. 2019 [28]	150/150	59.3/58.2	53.3/54.0	-	-	-	-	-	1.35/1.48	2.85/3.67	2.85/3.67	1.46/1.41	-	-	4.91/8.82	-	-	China
Wang JD, *et al*. 2017 [29]	384/582	58.4/59.2	-	25.3/28.4	23.7/25.4	136.8/138.5	76.4/76.5	13.5/29.0	3.98/4.73	1.35/1.14	2.96/3.65	1.43/1.69	12.2/30.6	-	-	-	-	China
Stabouli S, *et al*. 2020 [30]	24/18	12.0/16.0	11.0/13.0	20.0/20.2	-	116.4/117.8	73.2/71.5	-	3.41/3.89	-	-	-	-	88.5/90.1	-	-/8.22	69.8/57.5	Greece
Wang D, *et al*. 2019 [31]	100/266	64.2/65.7	59.0/57.1	-	-	-	-	-	5.00/4.30	1.20/1.10	2.90/2.80	1.60/1.80	-	-	-	-	-	China
Alvim R, *et al*. 2013 [32]	1867/136	41.8/52.7	46.9/39.7	25.7/29.6	23.6/27.4	125.3/134.9	81.6/85.2	24.4/45.0	5.16/5.49	1.21/1.10	3.50/3.60	1.41/2.24	24.0/45.0	-	-	-	-	Brazil
Dou LJ, *et al*. 2010 [33]	22/32	49.6/51.8	54.5/53.1	24.3/25.9	-	129.0/126.5	77.8/80.3	-	5.54/6.06	1.14/1.08	3.29/3.35	2.11/2.91	-	-	5.25/9.08	-	-	China
Zhu LY, *et al*. 2012 [34]	56/56	48.7/50.1	55.4/58.9	24.2/27.0	-	120.3/129.2	79.3/85.2	-	4.50/5.20	1.30/1.30	2.50/3.10	1.20/1.90	-	-	5.20/8.90	-	-	China
Guo Y, *et al*. 2011 [35]	65/162	70.5/72.9	60.0/59.9	22.9/24.1	-	121.0/128.0	71.0/76.0	-	4.46/4.49	1.37/1.24	2.67/3.55	1.45/1.67	-	-	5.43/6.77	5.52/6.76	-	China
Deng CL, *et al*. 2015 [36]	50/50	59.4/62.7	58.0/60.0	24.3/25.6	16.0/34.0	-	-	100.0/100.0	4.90/4.80	1.20/1.20	2.80/2.90	1.90/2.50	-	69.8/70.4	5.10/6.90	-	-	China
Yuan LX, *et al*. 2017 [37]	298/262	60.6/64.5	54.7/54.2	24.6/25.6	20.0/33.0	-	-	100.0/100.0	4.80/4.80	1.20/1.20	2.90/2.90	2.10/2.30	-	68.9/69.6	5.20/7.00	-	-	China
Chen S, *et al*. 2021 [38]	4584/584	49.4/63.3	40.3/50.7	24.0/24.9	19.6/22.8	123.1/132.3	76.6/78.0	-	4.80/4.85	1.23/1.13	2.76/2.72	1.74/2.05	-	-	-	-	-	China
Yue Q, *et al*. 2012 a [39]	40/40	56.7/59.9	50.0/45.0	25.6/25.7	22.5/20.0	125.6/139.2	80.8/89.8	-	4.30/4.78	1.24/1.16	2.48/2.53	1.46/2.04	-	-	-	-	-	China
Yue Q, *et al*. 2012 b [39]	40/40	58.1/58.9	57.5/52.5	24.9/26.1	22.5/22.5	135.5/145.8	89.1/93.3	-	4.20/4.40	1.15/1.19	2.28/2.33	1.95/2.14	-	-	-	-	-	China
Tian J, *et al*. 2018 [40]	100/100	57.6/58.5	58.0/58.0	22.6/26.3	-	132.4/125.6	84.3/79.6	27.0/62.0	-	-	-	-	19.0/56.0	62.1/65.9	-	5.32/6.56	-	China
Li MY, *et al*. 2013 [41]	78/41	49.2/53.0	44.9/43.9	26.1/270	-	137.5/142.8	83.5/86.5	100.0/100.0	4.84/4.91	1.22/1.23	3.04/3.00	1.55/2.45	-	-	5.37/7.43	-	68.15/65.24	China

“a”and “b” respectively represent two different sets of data in the same literature. BMI, body mass index; HDL, high-density lipoprotein; LDL, low-density lipoprotein; HbA1c, HemoglobinA1c; DM, diabetes mellitus.

### 3.2 Quality Assessment

Table 3 (Ref. [2, 3, 9, 10, 11, 12, 13, 14, 15, 16, 17, 18, 19, 20, 21, 22, 23, 24, 25, 26, 27, 28, 29, 30, 31, 32, 33, 34, 35, 36, 37, 38, 39, 40, 41, 43, 44, 45]) shows the quality assessments of the observational studies.

**Table 3.** **Quality assessments of the observational studies**.

Study	Selection	Comparability	Measurement	Total score
Bruno RM,*et al*. 2012 [2]	✩✩	✩✩	✩✩	6
Zhang M, *et al*. 2011 [3]	✩✩✩✩	✩✩	✩✩	8
Podgórski M, *et al*. 2019 [9]	✩✩✩	✩✩	✩✩	7
Coutinho MN, *et al*. 2018 [10]	✩✩	✩✩	✩✩	6
Cameron JD, *et al*. 2003 [11]	✩	✩	✩✩	4
Luneva EB, *et al*. 2022 [12]	✩✩✩✩	✩✩	✩✩	8
Toyoshima MTK, *et al*. 2023 [13]	✩✩	✩	✩✩	5
Loehr LR, *et al*. 2016 [14]	✩✩✩✩	✩✩	✩✩	8
Dybjer E, *et al*. 2018 [15]	✩✩✩✩	✩✩	✩✩	8
Gordin D, *et al*. 2016 [16]	✩✩✩		✩✩	5
Sciacqua A, *et al*. 2020 [17]	✩✩	✩✩	✩✩	6
Tuttolomondo A, *et al*. 2019 [18]	✩✩✩	✩✩	✩✩	7
Wiromrat P, *et al*. 2019 [43]	✩✩✩		✩✩	5
Metsämarttila E, *et al*. 2018 [19]	✩✩✩✩	✩✩	✩✩	8
Vallée A, *et al*. 2018 [20]	✩✩✩	✩✩	✩✩	7
van Mil SR, *et al*. 2018 [21]	✩✩✩		✩✩	5
Funck KL, *et al*. 2021 [22]	✩✩	✩✩	✩✩	6
Muhammad IF, *et al*. 2017 [23]	✩✩✩		✩✩	5
Martagón AJ, *et al*. 2022 [24]	✩✩✩✩	✩✩	✩✩	8
Zhou J, *et al*. 2018 [44]	✩✩	✩✩	✩✩	6
Tian CW, *et al*. 2015 [25]	✩✩✩✩	✩✩	✩✩	8
Liu LJ, *et al*. 2021 [26]	✩✩✩✩		✩✩	6
Zhang JX, *et al*. 2011 [27]	✩✩✩✩		✩✩	6
Li CS,* et al*. 2019 [28]	✩✩✩✩	✩✩	✩✩	8
Wang JD,* et al*. 2017 [29]	✩✩✩✩		✩✩	6
Stabouli S,* et al*. 2020 [30]	✩✩	✩✩	✩✩	6
Wang D,* et al*. 2019 [31]	✩✩	✩	✩✩	5
Alvim R,* et al*. 2013 [32]	✩✩✩✩	✩	✩✩	7
Dou LJ,* et al*. 2010 [33]	✩✩✩✩	✩✩	✩✩	8
Zhu LY,* et al*. 2012 [34]	✩✩✩✩		✩✩	6
Guo Y,* et al*. 2011 [35]	✩✩✩✩	✩✩	✩✩	8
Deng CL,* et al*. 2015 [36]	✩✩	✩	✩✩	5
Yuan LX,* et al*. 2017 [37]	✩✩		✩✩	4
Chen S,* et al*. 2021 [38]	✩✩✩✩	✩✩	✩✩	8
Yue Q,* et al*. 2012 [39]	✩✩	✩	✩✩	5
Tian J,* et al*. 2018 [40]	✩✩✩✩	✩	✩✩	7
Li MY,* et al*. 2013 [41]	✩✩	✩✩	✩✩	6

According to the rules of the items of the Newcastle–Ottawa Quality Assessment Scale [45], the quality of literature is scored. One star (✩) represents one point. The full score was 9 points: 7 to 9 points were deemed high-quality studies, 4 to 6 were medium-quality studies, and less than 4 were low-quality studies.

### 3.3 Primary Endpoints 

#### 3.3.1 cf-PWV between non-DM and DM Populations

**Fig. 2.**
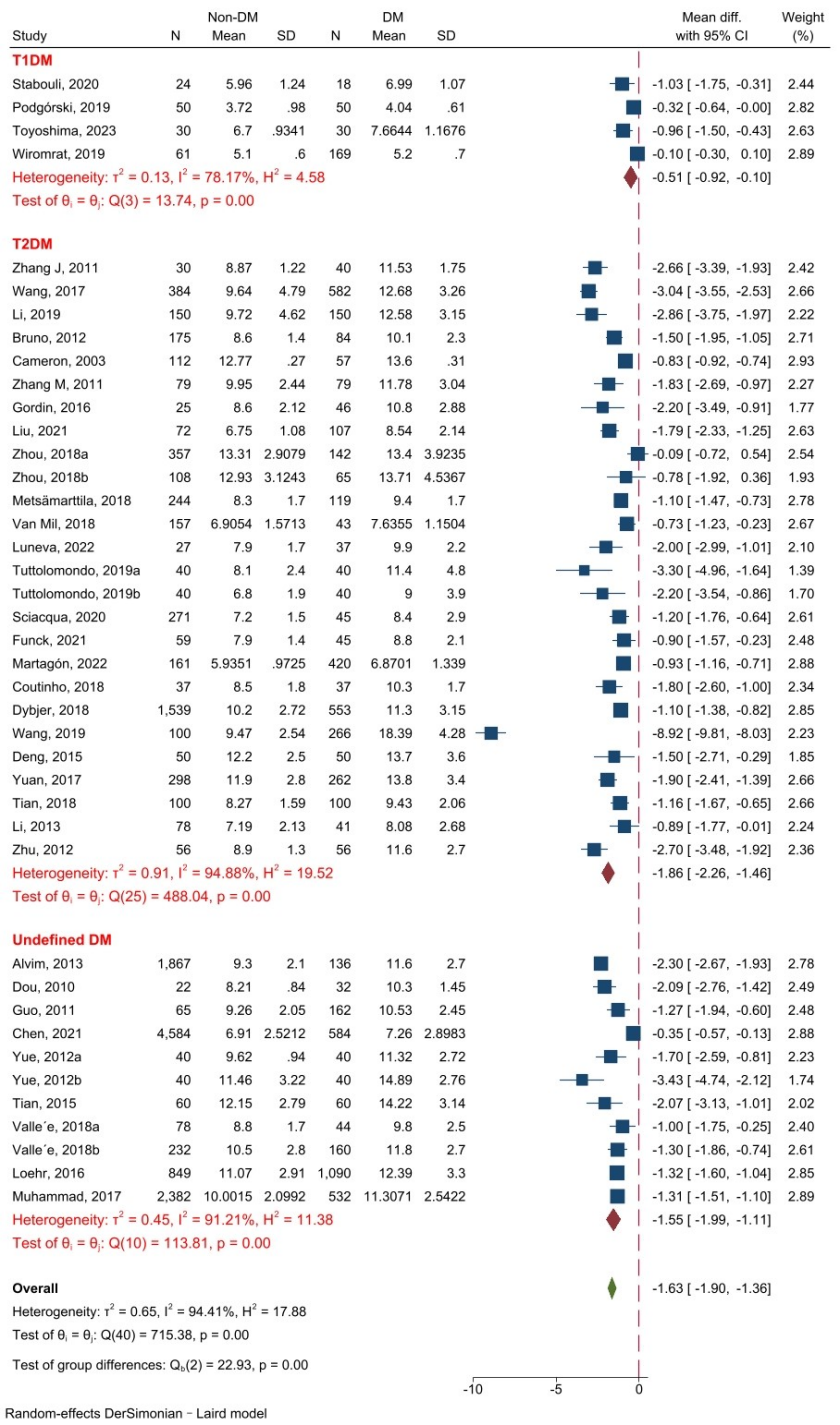
**Forest plot of cf-PWV comparison between non-DM and DM**. Abbreviations: T1DM, type 1 diabetes mellitus; T2DM, type 2 diabetes mellitus; cf-PWV, carotid–femoral pulse wave velocity; DM, diabetes mellitus.

**Fig. 3.**
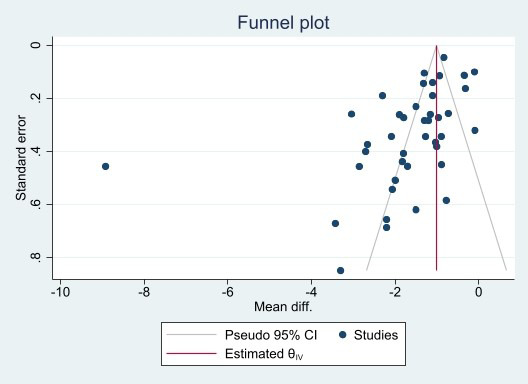
**Funnel plot for publication bias evaluation of studies concerning carotid–femoral pulse wave velocity**.

**Fig. 4.**
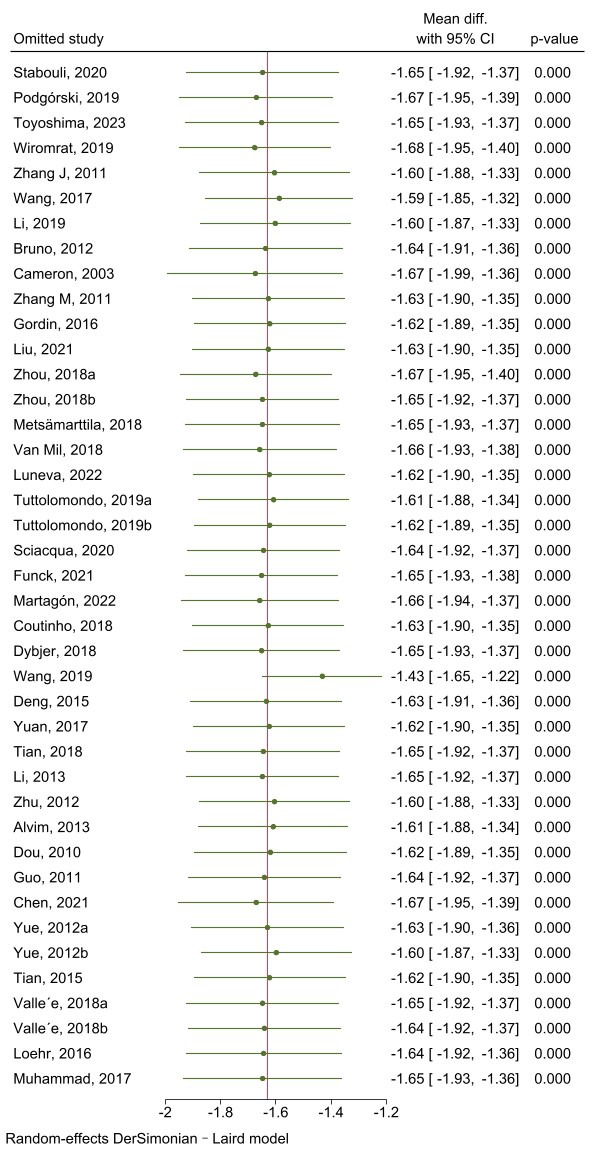
**Sensitivity analysis of the heterogeneity of studies concerning carotid–femoral pulse wave velocity**.

**Fig. 5.**
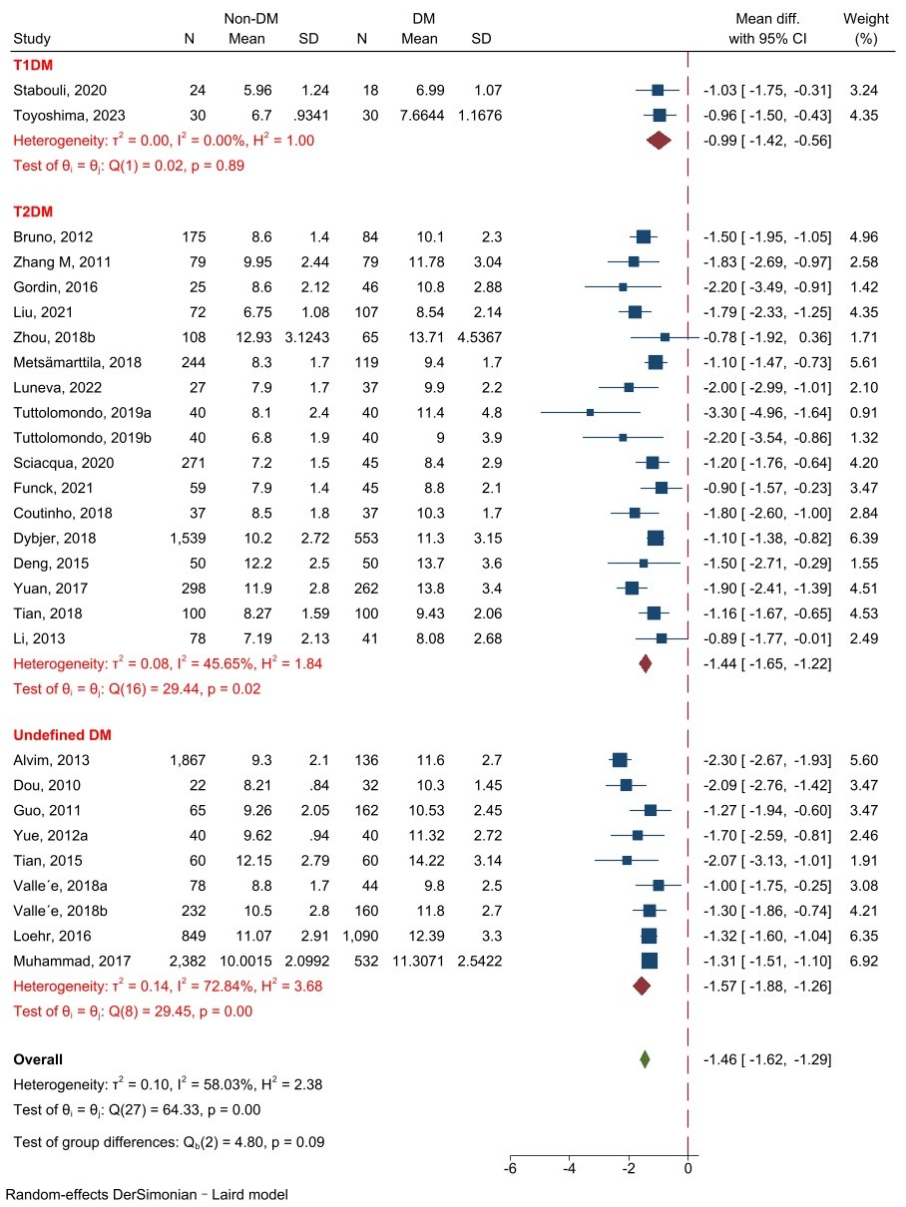
**Forest plot of cf-PWV comparison between the non-DM group and DM patients**. Abbreviations: T1DM, type 1 diabetes mellitus; T2DM, type 2 diabetes mellitus; cf-PWV, carotid–femoral pulse wave velocity; DM, diabetes mellitus.

A total of 37 studies involving 21,786 subjects were finally chosen to analyze cf-PWV between non-DM and DM samples. The analysis of the 37 studies revealed that DM patients had a significantly higher cf-PWV value than non-DM (z = –11.81, *p* < 0.001). Subgroup analysis revealed that all the T1DM, T2DM, and undefined DM subgroups were consistent with the overall effect. The heterogeneity was large (I^2^ = 94.41%, *p* < 0.001), so a random-effect model was used (Fig. 2). Analysis of publication bias showed no evident bias (Fig. 3). Sensitivity analysis showed that heterogeneity mainly came from the studies by Podgórski M*,et al*. 2019 [9], Wiromrat P, *et al*. 2019 [43], Zhang JX, *et al*. 2011 [27], Wang JD, *et al*. 2017 [29], Li CS, *et al*. 2019 [28], Cameron JD, *et al*. 2003 [11], Zhou J, *et al*. 2018a [44], van Mil SR, *et al*. 2018 [21], Martagón AJ, *et al*. 2022 [24], Wang D, *et al*. 2019 [31], Zhu LY, *et al*. 2012 [34], Chen S, *et al*. 2021 [38], Yue Q, *et al*. 2012b [39] (Fig. 4). After eliminating these studies, the heterogeneity was reduced (I^2^ = 58.03%, *p* < 0.001), and the subsequent result was consistent with the primary data (z = –16.84, *p* < 0.001) (Fig. 5).

#### 3.3.2 cf-PWV between Non-DM and Prediabetes

**Fig. 6.**
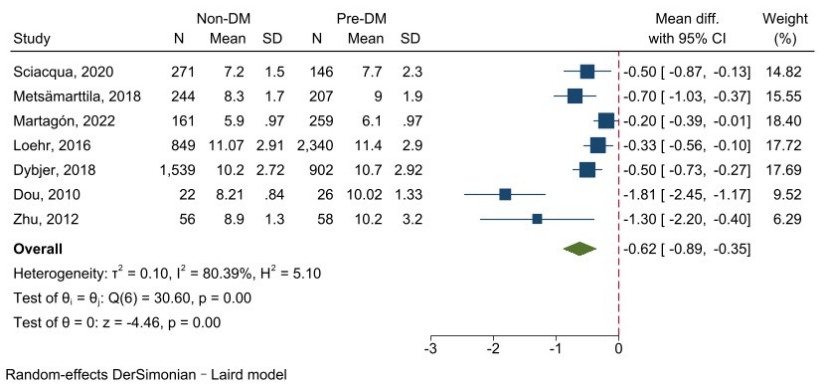
**Forest plot of cf-PWV comparison between the non-DM group and prediabetes patients**. Abbreviations: DM, diabetes mellitus; cf-PWV, carotid–femoral pulse wave velocity.

Of the 37 included studies, 7 reported cf-PWV comparison results between non-DM samples and prediabetes. The heterogeneity was large (I^2^ = 80.39%, *p* < 0.001), meaning the random-effect model was used. The overall results revealed prediabetes had a higher cf-PWV value than non-DM, and the difference was statistically significant (z = –4.46, *p* < 0.001) (Fig. 6).

### 3.4 Secondary Endpoint

#### 3.4.1 Carotid Intima-Media Thickness 

**Fig. 7.**
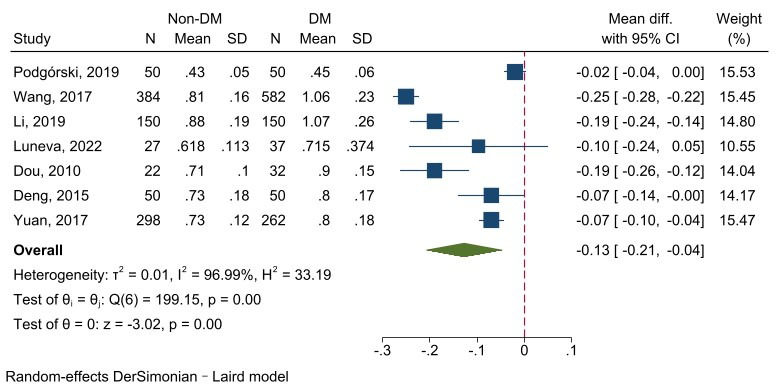
**Forest plot of cIMT comparison between the non-DM group and DM patients**. Abbreviations: DM, diabetes mellitus; cIMT, carotid intima-media thickness.

The testing of seven studies involving 2144 subjects revealed that DM patients had a significantly larger cIMT value than the non-DM (z = –3.02, *p* < 0.001). The heterogeneity was large (I^2^ = 96.99%, *p* < 0.001), meaning the random-effect model was used (Fig. 7).

#### 3.4.2 Carotid–Radial Pulse Wave Velocity

**Fig. 8.**
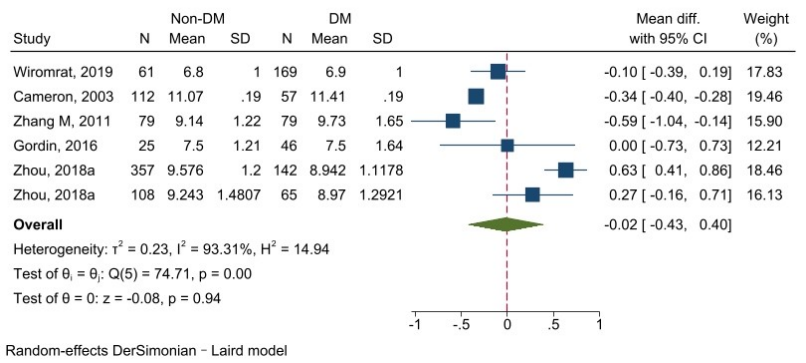
**Forest plot of cr-PWV comparison between the non-DM group and DM patients**. cr-PWV, carotid–radial pulse wave velocity; DM, diabetes mellitus.

The testing of five studies involving 1300 subjects revealed that DM patients had a higher cr-PWV value than the non-DM group, although the difference was not significant (z = –0.08, *p *= 0.94). The heterogeneity was large (I^2^ = 93.31%, *p* < 0.001), meaning the random-effect model was used (Fig. 8).

## 4. Discussion

AS is the earliest detectable indicator of structural and functional abnormalities in the arterial wall [46]. Currently, several studies have shown that AS has an important prognostic value as an independent predictor of CV disease and all-cause mortality [47, 48, 49]. Thus, it is increasingly recognized as a surrogate endpoint for CV disease [50]. A prospective observation of a large community-based sample of 20 years suggests that arterial stiffness is also important for the long-term outcomes of multiple health diseases, including hypertension, diabetes, chronic kidney disease, dementia, coronary heart disease, heart failure, and stroke [51]. To evaluate AS, there are several clinical and scientific methods and indicators, such as arterial compliance (compliance) [52], arterial dilation (distensibility) [53], and elastic modulus (E) [54]. Among them, the PWV has been recognized as the most effective and noninvasive method [55]. PWV refers to the velocity of the pulse pressure wave generated by the arterial vascular tree, which is accurate, reproducible, and easy to measure [56].

There is no consensus on the association of AS with the risk of diabetes. Some studies showed a positive association between AS and diabetes, demonstrating that the PWV of prediabetic and diabetic patients was higher than in those with normal fasting blood glucose, and even nondiabetic individuals with simple insulin resistance still had arterial stiffness [57, 58]. Nevertheless, some studies failed to demonstrate this significant association [5, 43, 44]. A Brazilian study found that the PWV difference of people with diabetes compared with nondiabetics was not significant [32].

The latest meta-analyses studying the relationship between AS and DM were performed in 2015 and 2019 [59, 60]. Their subjects were DM and normal glucose samples, meaning prediabetes was not included. They used cIMT and ba-PWV as the indicators of AS and enrolled a limited number of studies containing cf-PWV. Indeed, cIMT is a traditional index for evaluating AS, which reflects the morphological changes in the local vascular wall of the carotid artery but cannot directly reflect the overall condition of a large artery, and the morphological changes often lag behind the functional changes [61]. Although ba-PWV avoids these limitations, the measurement area includes the central artery and parts of the peripheral artery and cannot clearly reflect the specific segments in which the artery changes occur [62]. It has been shown that central arterial stiffness rather than peripheral arterial stiffness can predict the risk level of future cardiovascular disease, and PWV of the central artery might have a more significant value [50]. Additionally, aortic stiffness can predict future cardiovascular events and mortality, even after accounting for other established cardiovascular risk factors [63]. Kimoto *et al*. [64] found that PWV in four arterial areas in patients with T2DM was higher than in healthy subjects, and the effect of diabetes on heart–carotid and heart–femoral PWV was greater than heart–brachial and femoral–ankle PWV, suggesting that T2DM has more influence on aortic PWV than peripheral arteries. However, the evaluation of aortic stiffness did not use cf-PWV [64]. The cf-PWV was initially used in 2007 by the European Society of Hypertension and the Society of Cardiology as the gold standard for assessing arterial stiffness and as a tool for assessing subclinical target organ injury [4]. However, due to its relatively complex measurement and acquisition techniques in the early stages, it has yet to be widely used in clinical practice. Recently, with the continuous development of instruments and technologies, cf-PWV has been more frequently used in clinical practice. In 2023, the ESH guidelines for managing arterial hypertension identified cf-PWV >10 m/s as one of the important markers of hypertension-related arterial injury [65]. As cf-PWV was gradually proven more valuable in predicting future cardiovascular events, we performed this meta-analysis to explore the association between DM and cf-PWV. We conducted a comprehensive literature search and inclusion process to analyze the differences in cf-PWV between individuals with DM and non-diabetic controls. We also collated and analyzed the prediabetes population data in these studies. cIMT and cr-PWV in the included studies were also analyzed as the secondary endpoints. To the best of our knowledge, this is the first meta-analysis using cf-PWV to explore this issue.

The results from the 37 included studies revealed that patients with DM (both type 1 and type 2) had a higher cf-PWV, and the difference was statistically significant. Seven of the studies, including patients with prediabetes, also yielded the same results. Similarly, in the secondary endpoint, we also found that DM had a higher cIMT, which is consistent with the results of previous studies. However, when we analyzed the other secondary endpoints, the results showed that patients with diabetes had higher cr-PWV than those without diabetes, although the difference was not significant. The cr-PWV reflects the peripheral arterial stiffness and, specifically, the arterial stiffness of the upper limb vessels. Although diabetes involves peripheral nerve and vascular lesions, it is unclear whether the upper limb arterial stiffness is worse due to the limited literature on this subject and sampling errors. The heterogeneity of the five studies was large. Some studies had contradictory conclusions, which might be attributed to sampling errors and the limitations of cr-PWV in evaluating artery stiffness.

## 5. Limitations and Suggestions

Our study also has some limitations. There was strong heterogeneity among the included literature, and although both T1DM and T2DM were included in the study, each study had different inclusion and exclusion criteria, as well as different physical conditions. Various techniques were used to measure PWV, including pressure wave methodology and ultrasonic Doppler methods, and the consistency between the methods was not accurately verified. Furthermore, our study included limited literature on prediabetes and did not analyze the extent of arterial stiffness in the lower extremities of diabetic patients. In addition, different methodological considerations may have also impacted the results. Most of the included studies are cross-sectional observational studies. Thus, more longitudinal studies involving interventions should be conducted in the future.

## 6. Conclusions

In summary, DM (both type 1 and type 2) or prediabetes were associated with higher cf-PWV compared to those with normal blood sugar. Higher AS means a higher risk of cardiovascular complications and death. Individuals at high risk of both diseases can be identified by the early measurement of cf-PWV, following which appropriate interventions can be actively adopted to reduce the risk of cardiovascular complications in these high-risk patients.

## Availability of Data and Materials

Template data collection forms, data extracted from included studies, data used for all analyses, and analytic code in the review are publicly available.

## Abbreviations

AS, aortic stiffness; AIx, augmentation index; cIMT, carotid intima-media thickness; DM, diabetes mellitus; T1DM, type 1 diabetes; T2DM, type 2 diabetes; PWV, pulse wave velocity; cf-PWV, carotid–femoral PWV; ba-PWV, brachial–ankle PWV; cr-PWV, carotid–radial PWV; MD, mean difference; CI, confidence interval.

## Supplementary Material

Supplementary material associated with this article can be found, in the online version, at https://doi.org/10.31083/j.rcm2509339.
